# The Antivivisection Bill in the U. S. Senate

**Published:** 1897-08

**Authors:** 


VETERINARIANS will bear in mind that Senate Bill No. 1063 still remains on the calendar of that body waiting a favorable opportunity to be called up for passage. This bill strikes right at the root of all original investigations of the Bureau of Animal Industry, and would likely be followed by similar efforts in many States, and thus would halt all progress in human and comparative medical science.

